# Autonomously Replicating Linear Plasmids That Facilitate the Analysis of Replication Origin Function in *Candida albicans*

**DOI:** 10.1128/mSphere.00103-19

**Published:** 2019-03-06

**Authors:** Swati Bijlani, Mathuravani A. Thevandavakkam, Hung-Ji Tsai, Judith Berman

**Affiliations:** a School of Molecular Cell Biology and Biotechnology, Tel Aviv University, Ramat Aviv, Israel; b Department of Cell Biology, Johns Hopkins School of Medicine, Baltimore, Maryland, USA; Carnegie Mellon University

**Keywords:** *CaURA3*, linear plasmids, replication, replication origins, telomere repeats

## Abstract

The ability to generate autonomously replicating plasmids has been elusive in Candida albicans, a prevalent human fungal commensal and pathogen. Instead, plasmids generally integrate into the genome. Here, we assessed plasmid and transformant properties, including plasmid geometry, transformant colony size, four selectable markers, and potential origins of replication, for their ability to drive autonomous plasmid maintenance. Importantly, linear plasmids with terminal telomere repeats yielded many more autonomous transformants than circular plasmids with the identical sequences. Furthermore, we could distinguish (by colony size) transient, autonomously replicating, and chromosomally integrated transformants (tiny, medium, and large, respectively). Candida albicans *URA3* and a heterologous marker, *ARG4,* yielded many transient transformants indicative of weak origin activity; the replication of the plasmid carrying the heterologous *LEU2* marker was highly dependent upon the addition of a *bona fide* origin sequence. Several *bona fide* chromosomal origins, with an origin fragment of ∼100 bp as well as a heterologous origin, *panARS*, from Kluyveromyces lactis, drove autonomous replication, yielding moderate transformation efficiency and plasmid stability. Thus, C. albicans maintains linear plasmids that yield high transformation efficiency and are maintained autonomously in an origin-dependent manner.

**IMPORTANCE** Circular plasmids are important tools for molecular manipulation in model fungi such as baker’s yeast, yet, in Candida albicans, an important yeast pathogen of humans, prior studies were not able to generate circular plasmids that were autonomous (duplicated without inserting themselves into the chromosome). Here, we found that linearizing circular plasmids with sequences from telomeres, the chromosome ends, allows the plasmids to duplicate and segregate in C. albicans. We used this system to identify chromosomal sequences that facilitate the initiation of plasmid replication (origins) and to show that an ∼100-bp fragment of a C. albicans origin and an origin sequence from a distantly related yeast can both function as origins in C. albicans. Thus, the requirements for plasmid geometry, but not necessarily for origin sequences, differ between C. albicans and baker’s yeast.

## Abstract (precis)

Circular plasmids are important tools for molecular manipulation in model fungi such as baker’s yeast, yet, in Candida albicans, an important yeast pathogen of humans, prior studies were not able to generate circular plasmids that were autonomous (duplicated without inserting themselves into the chromosome). Here, we found that linearizing circular plasmids with sequences from telomeres, the chromosome ends, allows the plasmids to duplicate and segregate in C. albicans. We used this system to identify chromosomal sequences that facilitate the initiation of plasmid replication (origins) and to show that an ∼100-bp fragment of a C. albicans origin and an origin sequence from a distantly related yeast can both function as origins in C. albicans. Thus, the requirements for plasmid geometry, but not necessarily for origin sequences, differ between C. albicans and baker’s yeast.

## INTRODUCTION

Plasmids are autonomously replicating extrachromosomal elements that facilitate molecular studies in bacteria as well as in yeasts and other fungi (1). Some yeast species carry circular plasmids (e.g., 2µ in Saccharomyces cerevisiae [2]) or linear plasmids (e.g., killer plasmids in Kluyveromyces lactis [3] and mitochondrial plasmids in Fusarium oxysporum [4, 5]). Plasmid replication requires, among other components, an origin DNA sequence to which the origin recognition complex (ORC) binds. Origins of replication initiation (ORIs) on chromosomes and plasmids appear to have different sequence requirements in different yeast species (6). In S. cerevisiae, autonomously replicating sequences (ARSs; ORIs able to drive plasmid replication) are modular, requiring a minimum of 100 bp that includes an 11-bp ARS consensus sequence (ACS) (7–9) and a T-rich “B element” (10, 11). In most other organisms, the DNA requirements for centromere and ORI function are less well defined: K. lactis requires a 50-bp ACS that is necessary and sufficient for ARS activity (12), and Schizosaccharomyces pombe has no specific ARS consensus but requires a region of >500 bp with multiple A-T hook motifs that binds the ORC (13–15).

In Candida albicans, a common human fungal commensal and an opportunistic pathogen, ORIs have been only partially characterized. C. albicans origins, like those of S. pombe and higher eukaryotes, have longer and less well defined DNA motifs (16). Prior work with S. cerevisiae identified ARSs based on their high transformation efficiency (17, 18). Early studies found that *Sc*ARS plasmids with circular or linear geometry could be maintained autonomously for some time (19, 20). Work in C. albicans identified a few sequences that conferred high transformation efficiency on circular plasmids (21–27). However, either the resulting transformants were highly unstable (transient transformants) or the plasmid rapidly integrated into the genome (integrants). The *CaURA3* marker used in many of these studies was later found to have an intrinsic weak ARS activity (28), and there was no direct evidence that replication initiated from the inserted sequences.

We previously used a machine learning approach to identify proposed-origins (pro-ORIs) based on ORC binding activity and nucleosome occupancy patterns (28). Four pro-ORIs were shown to be *bona fide* origins that produced replication bubble structures on nondenaturing 2-dimensional (2-D) DNA gels, thereby providing direct evidence of ORI function (28). Importantly, all four *bona fide* ORIs also drove plasmid replication on linear (but not circular) plasmids derived from circles carrying long inverted telomere (TEL) repeats separated by a spacer sequence that is cleaved to linearize the plasmid (29). These large plasmids with inverted telomere sequences could work well but were prone to rearrangement during propagation of the circular precursor plasmid in Escherichia coli.

Here, we compared circular and linear plasmids in C. albicans that rely on *bona fide* ORIs for their maintenance. Linear plasmids were constructed from circles *de novo* by PCR with primers bearing telomeric repeats prior to transformation. Linear plasmids consistently had higher transformation efficiency, larger numbers of autonomous transformants, and higher mitotic stability than analogous circular plasmids. Transformant colony size was a clear reflection of plasmid stability, with tiny colonies indicative of unstable, transient transformants; medium-size colonies indicative of autonomous transformants with moderate stability levels; and large, smooth colonies indicative of integrants, in which plasmid was inserted at chromosomal positions. We also tested four markers including *CaURA3* and *CaHIS1* as well as heterologous markers *CdARG4* and *CmLEU2* (30), which all had different levels of origin-dependent transformation efficiency and maintenance. Finally, we tested *bona fide* ORIs (28) as well as origin fragments and heterologous origin sequences and found that an ∼100-bp ORI fragment and a K. lactis *panARS* (31) have moderate origin activity in C. albicans.

## RESULTS

### Circular *CaURA3* plasmids with and without ORIs.

In this study, we investigated the outcomes of transformations with plasmids that differed by selection markers, geometry (circular versus linear), and replication origins. We compared the transformation parameters, including transformation efficiency (number of transformants/µg of DNA), size of the transformant colonies (tiny [<0.4 mm], medium [0.4 to 1.6 mm], and large [>1.6 mm]; Table 1), mitotic stability (proportion of cells that retain the plasmid under selection), and plasmid loss rate (rate of plasmid loss per generation in the absence of selection). We used a set of isogenic strains that differed primarily in the relevant auxotrophy.

**TABLE 1:** Properties of different types of transformants obtained with circular and linear plasmids

Type of colony	Size category	Size (mm in diam)	Lag time (min)	Doubling time (min)	Relevant plasmid(s)	Mitotic stability (%)
Transient	Tiny	≤0.4	174 ± 5	856 ± 3	pCir/pLin-*CaURA3* (±*ORI410*), pCir/pLin-*CaHIS1* (±*ORI410*), pCir/pLin-*CmLEU2* (±*ORI410*), pCir/pLin-*CdARG4* (±*ORI410*)	0
Autonomous	Medium	0.4–1.6	30 ± 10	140 ± 50	pCir-*CaHIS1* (±*ORI410*), pCir-*CdARG4* (±*ORI410*), pCir-*CmLEU2* (+*ORI410*)	≤10
	Medium	0.4–1.6	28 ± 6	42 ± 27	pLin-*CaURA3* (±*ORI410*), pLin-*CaHIS1* (±*ORI410*), pLin-*CdARG4* (±*ORI410*), pLin-*CmLEU2* (±*ORI410*)	10–40
Integrant	Large	1.6–2.2	19 ± 2	25 ± 3	pCir/pLin-*CaHIS1* (±*ORI410*)	80–100

Overall, across the markers and plasmids tested, three types of transformant colonies were evident. Tiny colonies that could not be maintained on selection (see Fig. S1A and B in the supplemental material) with undetectable plasmid retention (mitotic stability, ∼0) indicative of rapid plasmid loss were defined as transient transformants (referred to as transients here) (Table 1). Large, round colonies with short lag time and doubling time (Table 1; Fig. S1C) were defined as integrants based on their high stability under selection (mitotic stability, ∼80 to 100%). Medium colonies (mitotic stability, 1 to 80%) that grew, albeit less well than integrants under selection, with comparatively longer lag and doubling times (Table 1; Fig. S1D), were defined as autonomously replicating transformants (ARS-transformants), assuming that replicating plasmids can be maintained under selection and lost in the absence of selection. Accordingly, colony size can reliably predict the mitotic stability of plasmids and used as a proxy for the number of different transformant types.

10.1128/mSphere.00103-19.1 FIG S1 Growth curves of transients obtained with pCir-*CaURA3*-*ORI410* (A), transients obtained with pCir-*CdARG4*-*ORI410* (B), integrants obtained with pCir-*CdARG4*-*ORI410* (C), ARS-transformants obtained with pCir-*CdARG4*-*ORI410* (D), and ARS-transformants obtained with pLin-*CdARG4*-*ORI410* (E and F). The growth curves are representative of one colony of each type (the growth parameters averaged for three colonies of each type are listed in Table 1). Download FIG S1, PDF file, 0.08 MB. Copyright © 2019 Bijlani et al. 2019 Bijlani et al. This content is distributed under the terms of the Creative Commons Attribution 4.0 International license.

**FIG 1:**
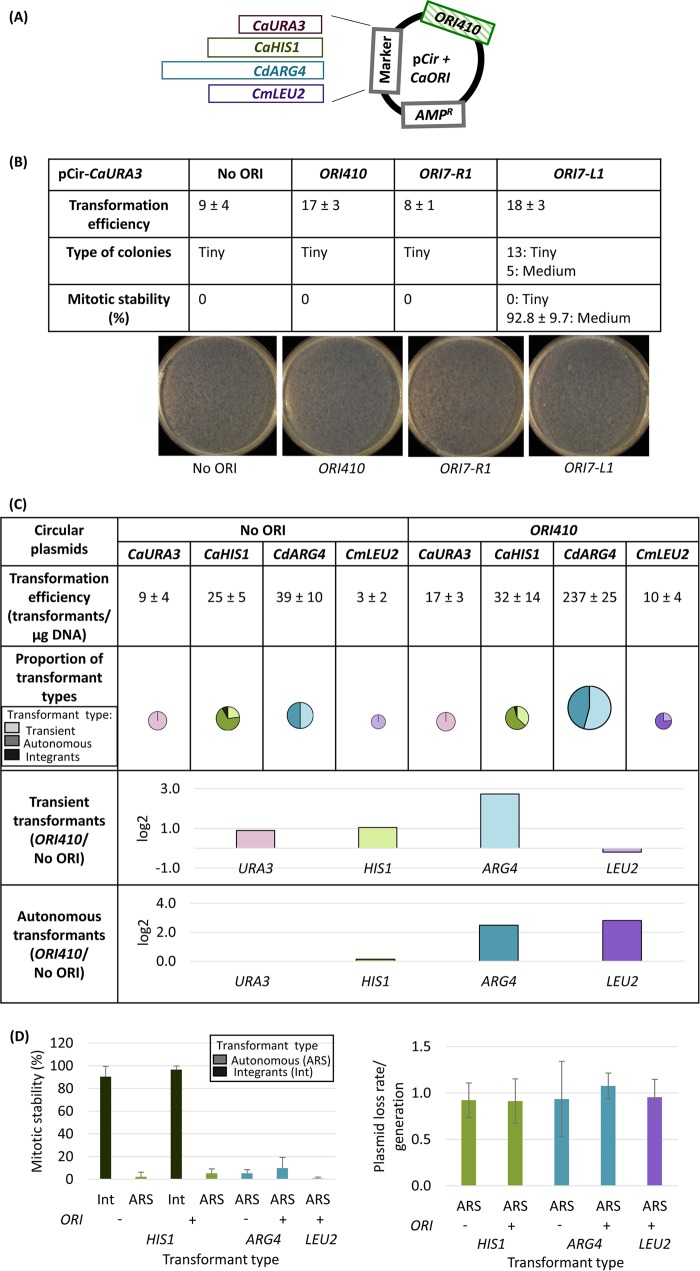
(A) Map of a circular plasmid showing relative position of selection markers (*CaURA3*, *CaHIS1*, *CdARG4*, and *CmLEU2*) and ORI sequence. (B) pCir-*CaURA3* plasmid with different origin sequences transformed in C. albicans BWP17: transformation efficiency, types of colonies, and their mitotic stability. The transformation efficiency is an average from three independent experiments. (C) Comparison of circular plasmids carrying different selection markers with and without *ORI410*: transformation efficiency, proportion of different types of transformants, and log_2_ value of the ratio of average number of transient or autonomous transformants with ORI to that without ORI (*ORI410*/No ORI). *CaURA3*, *CaHIS1*, *CdARG4*, and *CmLEU2* plasmids were transformed in C. albicans BWP17, SN76, SN76, and SN152, respectively. Different markers are represented by different colors, and different types of transformants are represented by various shades of a color (lightest shade representing transients, intermediate shade representing ARS-transformants, and darkest shade representing integrants). The transformation efficiency is an average from three independent experiments. The size of circles showing proportion of transformant types is an approximation of transformation efficiency. (D) Mitotic stability (%) of integrants and ARS-transformants and plasmid loss rate/generation for ARS-transformants obtained with different circular plasmids with and without *ORI410*. The data represent the average from three independent colonies of each type. Int, integrants; ARS, ARS-transformants.

To test the hypothesis that *bona fide* ORIs drive the autonomous replication of plasmids in C. albicans, we first constructed circular plasmids with the *CaURA3* marker similar to those from prior studies (22, 23, 25–27) with and without *bona fide ORI410* (28) (Fig. 1A). Transformation efficiency with and without *ORI410* was relatively modest (17 and 9 transformants/µg DNA, respectively [Fig. 1B]). Importantly, all selected colonies were tiny (<0.4 mm), with and without inclusion of *ORI410* (Fig. 1B). The tiny colonies did not grow upon restreaking or when seeded into liquid cultures (Fig. S1A), which is a characteristic of transients. Thus, as in several prior studies (22, 23, 25, 27), circular *CaURA3* plasmids were not maintained autonomously.

Because this result conflicts with the claim that two sequences, *ORI7-R1* and *ORI7-L1*, drive the replication of a circular *CaURA3* plasmid (26), we constructed plasmids with these sequences in pCir-*CaURA3.* Both of them had modest transformation efficiency (8 and 18 transformants, respectively; Fig. 1B). We obtained only transients for *ORI7-R1* with similar transformation efficiency as the no-ORI plasmid; *ORI7-L1* gave twice as many transients as no-ORI plasmid and produced a small number of stable transformants (Fig. 1B), indicating that they integrated into the genome. Thus, neither of the two *CEN7* flanking sequences yielded autonomous transformants in the context of a circular *CaURA3* plasmid (Fig. 1B), consistent with the poor performance of pCir-*CaURA3-ORI410*. Similar results for pCir-*CaURA3* with *ORI410*, *ORI7-L1*, and *ORI7-R1* were also evident in a second strain background (Table S1).

10.1128/mSphere.00103-19.7 TABLE S1 Transformation efficiency (average from three independent experiments) of pCir-*CaURA3* with different origin sequences in C. albicans BWP17 and SN76. Download Table S1, PDF file, 0.1 MB. Copyright © 2019 Bijlani et al. 2019 Bijlani et al. This content is distributed under the terms of the Creative Commons Attribution 4.0 International license.

### Comparison of different selectable markers on circular plasmid.

We next asked if the C. albicans *HIS1* (*CaHIS1*) marker would show better transformation efficiency and mitotic stability than *CaURA3*, with the goal of obtaining ARS-transformants. However, the *CaHIS1* plasmid yielded small numbers of transformants (32 and 25, with and without *ORI410*, respectively), with a modest increase (∼25%) in transformation efficiency attributable to *ORI410* (Fig. 1C). pCir-*CaHIS1* ARS-transformants had mitotic stability of <5% and plasmid loss rates of ∼0.9 (Fig. 1D), indicating that they were autonomous but highly unstable. Thus, in addition to transients and integrants (analyzed in more detail below), pCir-*CaHIS1* produced a small number of ARS-transformants—a group not detected with pCir-*CaURA3* (Fig. 1C).

Because heterologous markers are less likely to integrate into the genome, we tested the *CmLEU2* marker from Candida maltosa and the *CdARG4* marker from Candida dubliniensis (30). With the addition of *ORI410*, transformation efficiency of *CmLEU2* was increased by ∼3 times (Fig. 1C), and most of them were ARS-transformants with mitotic stability of <5% (Fig. 1D) compared to only transients without *ORI410*; no large colonies were detected. Thus, *CmLEU2* produced a small number of ARS-transformants with low mitotic stability upon addition of *ORI410*.

In contrast, *CdARG4* had a 5-fold-higher transformation efficiency with *ORI410* on the plasmid relative to that without the ORI, ∼50% being ARS-transformants (Fig. 1C) that had mitotic stability of ∼10% for those with *ORI410* and ∼5% for those without the ORI (Fig. 1D). Thus, *CdARG4* with *ORI410* yielded more than 100 ARS-transformants/µg of DNA, with an improved mitotic stability (but with the loss rate remaining quite high [Fig. 1D]). However, while *ORI410* was required for high transformation efficiency and improved mitotic stability, it was not required for some autonomous plasmid replication. We suggest that the *CdARG4* sequence might enable a weak ORI to form on the plasmid (discussed below). Thus, for circular plasmids with all four selectable markers tested, the inclusion of an origin was not sufficient to produce relatively stable autonomously replicating plasmids (low mitotic stability and high loss rate). This indicates that a heterologous marker can drive autonomous replication of a circular plasmid with rare integration events, but they are lost at high frequency.

We also asked if autonomous plasmids were detectable in DNA extracts from the medium colonies (low mitotic stability and high loss rate). Indeed, Southern blotting of DNA from medium colonies (Fig. S2A) detected bands with similar electrophoretic mobility as that of naked circular plasmids. In contrast, in the DNA from a pCir-*CdARG4*-*ORI410* large colony with high mitotic stability (presumed integrant), a larger band was detected along with autonomously replicating plasmid (Fig. S2A), indicating integration in some cells in a population. This is consistent with the idea that large colonies contain integrated plasmid and medium colonies contain autonomously replicating plasmids. Moreover, analysis of the *CaHIS1* integrants found gene replacement at the native locus by single crossover (Fig. S3A).

10.1128/mSphere.00103-19.2 FIG S2 Southern blot of ApaI*-*digested DNA isolated from different transformants and probed with *AMP^R^*. (A) Circular plasmids: pCir-*CaHIS1*-*ORI410* ARS-transformants (lanes 1 and 2), pCir-*CdARG4*-*ORI410* integrant (lane 3), pCir-*CdARG4-ORI410* ARS-transformants (lanes 4 and 5), pCir-*CaHIS1*-*ORI410* control plasmid (lane 6), and pCir-*CdARG4*-*ORI410* control plasmid (lane 7). All ARS-transformants gave a band corresponding to their control plasmid, indicating the existence of autonomously replicating plasmids. (B) Linear plasmids: pLin-*CaHIS1*-*ORI410* and ARS-transformants with mitotic stability of 46% and 63% (lanes 1 and 2), pLin-*CdARG4*-*ORI410* ARS-transformant with mitotic stability of 44% (lane 3), pLin-*CaHIS1*-*ORI410* control plasmid (lane 4), and pLin-*CdARG4*-*ORI410* control plasmid (lane 5). pLin-*CaHIS1*-*ORI410* plasmids showed integration, whereas the pLin-*CdARG4*-*ORI410* ARS-transformant gave a band corresponding to its control plasmid, indicating an autonomously replicating plasmid. Int, integrants; ARS, ARS-transformants. Download FIG S2, PDF file, 0.3 MB. Copyright © 2019 Bijlani et al. 2019 Bijlani et al. This content is distributed under the terms of the Creative Commons Attribution 4.0 International license.

10.1128/mSphere.00103-19.3 FIG S3 (A) Schematic showing integration of pCir-*CaHIS1*-*ORI410* into the genome by single crossover at either A or B. The integration was confirmed in three independent integrants by different primer sets indicated in the figure along with the size of PCR product expected. (B) Schematic showing integration of pLin-*CaHIS1*-*ORI410* into the genome by a double-crossover or gene conversion event. The integration was confirmed in three independent integrants by different primer sets indicated in the figure along with the size of the PCR product expected. M, marker; C, parent strain C. albicans SN76; Int, integrants. Download FIG S3, PDF file, 0.2 MB. Copyright © 2019 Bijlani et al. 2019 Bijlani et al. This content is distributed under the terms of the Creative Commons Attribution 4.0 International license.

### Linear plasmids with telomere ends are maintained autonomously.

**FIG 2:**
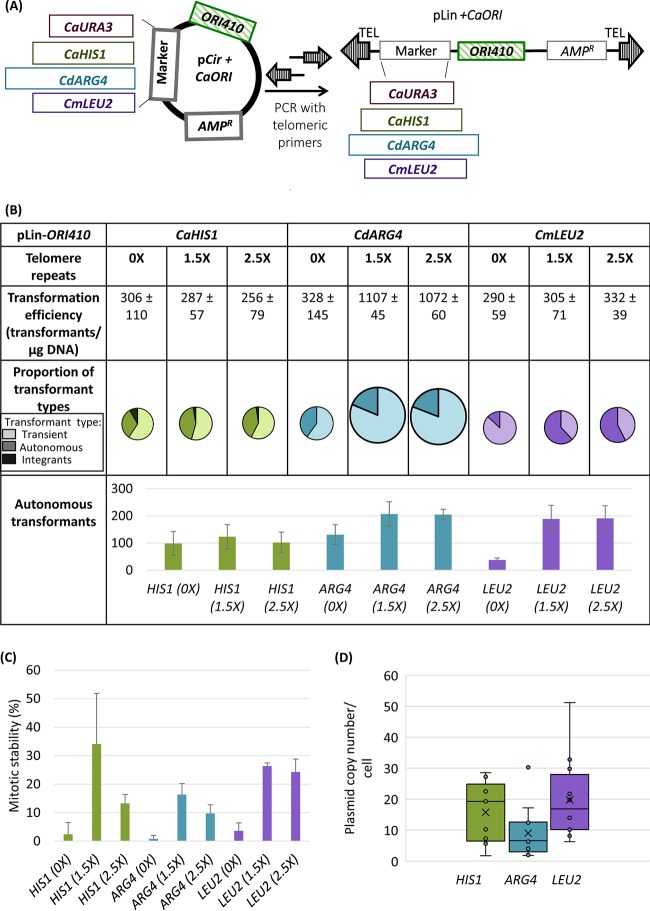
(A) Schematic of construction of linear plasmid using primers with telomeric repeats at their ends. (B) Comparison of linear plasmids carrying different selection markers and *ORI410* with 0×, 1.5×, and 2.5× telomere repeats at its ends: transformation efficiency, proportion of different types of transformants, and the number of autonomous transformants. *CaHIS1* and *CdARG4* plasmids were transformed in C. albicans SN76, and *CmLEU2* plasmids were transformed in C. albicans SN152. Different markers are represented by different colors, and different types of transformants are represented by various shades of a color (lightest shade representing transients, intermediate shade representing ARS-transformants, and darkest shade representing integrants). The transformation efficiency and number of autonomous transformants are an average for three independent experiments. The size of circles showing proportion of transformant types is an approximation of transformation efficiency. (C) Mitotic stability (%) of ARS-transformants obtained with different linear plasmids with 0×, 1.5×, and 2.5× telomere repeats. The data represent the average for three independent ARS-transformants. (D) A box plot representing copy number variations of linear plasmids with *CaHIS1*, *CdARG4*, or *CmLEU2* and *ORI410*. The data represent the average copy number of nine independent ARS-transformants (accounting for mitotic stability). In the box plot, dots represent different samples, crosses represent mean values, and the horizontal lines represent the medians.

Since circular plasmids did not yield high transformation efficiency and high mitotic stability for ARS-transformants, we constructed and transformed linear plasmids, which are known to replicate autonomously in some fungal model organisms (4, 32–35). Since classical methods of producing linear plasmids (29) used for monitoring origin function in C. albicans (28) proved challenging, we designed a new approach in which linear plasmids were constructed from circular plasmids by PCR (details in Materials and Methods) (Fig. 2A) and transformed directly into C. albicans.

Because telomere sequences are not necessary to be added to linear DNA during transformation in some fungal models (4, 32–34), we asked if the presence and the length of the telomere repeats (34-nt versus 57-nt TEL, i.e., 1.5× versus 2.5× of a single 23-nt *C. albicans* TEL repeat [36]) affect transformation parameters. Linear plasmids without TEL repeats had a transformation efficiency of ∼300/µg for all three markers tested (*CaHIS1*, *CdARG4*, and *CmLEU2*) with the majority being transients (Fig. 2B). The *CaHIS1* linear plasmid without telomere repeats resulted in an increased number of total and ARS-transformants but also an increase in integration events over the corresponding circular plasmid (Fig. 2B versus Fig. 1C; *P* ≤ 0.025 and *P* ≤ 0.044 for total and ARS-transformants, respectively, using Student’s *t* test). Notably, the *CmLEU2* linear plasmid without telomere repeats resulted in a much higher transformation efficiency and more ARS-transformants (∼50/µg DNA) than the corresponding circular plasmid (<10/µg DNA [Fig. 2B versus Fig. 1C]; *P* ≤ 0.007 and *P* ≤ 0.003 for total and ARS-transformants, respectively, using Student’s *t* test). The *CdARG4* plasmid was an exception yielding similar transformation efficiencies in circular and linear plasmids without telomeres (Fig. 2B versus Fig. 1C). However, all of the ARS-transformants obtained had low mitotic stability (<5%) (Fig. 2C) with an irregular colony shape, indicating that they were not readily maintained in the autonomous state, and higher proportions of cells failed to divide in the colony under selection conditions.

Adding TEL repeats to linear plasmids increased the number of ARS-transformants for both *CdARG4* and *CmLEU2* plasmids, compared to those without TEL repeats (Fig. 2B; *P* ≤ 0.043 and *P* ≤ 0.018 for *CdARG4* and *CmLEU2* ARS-transformants, respectively, using Student’s *t* test). In contrast, TEL sequence addition increased the transformation efficiency only for *CdARG4* plasmids among the three markers tested. Furthermore, adding TEL repeats increased the mitotic stability of ARS-transformants by 2- to 6-fold (mitotic stability, ∼10 to 35%) for all three markers. Thus, relative to circular plasmids, linearized plasmids with terminal TEL repeats produced more ARS-transformants with higher mitotic stability (Fig. 2B and C), and the ARS-transformants displayed shorter lag time and doubling time (Table 1; see also Fig. S1E and F). In contrast, when 1.5× TEL sequence was included on circular plasmids, there was no significant change in any of the transformation parameters relative to the corresponding circular plasmids (Fig. S4A and B). Thus, it is likely the linear geometry of the plasmids, along with the inclusion of telomere sequence, that resulted in an increase in ARS-transformants and mitotic stability (see Discussion). Since we found no obvious advantage to including 2.5× versus 1.5× TEL repeats, we used plasmids linearized with the 1.5× TEL repeats in all subsequent studies.

10.1128/mSphere.00103-19.4 FIG S4 (A) Comparison of circular plasmid, linear plasmid (with 1.5× TEL repeats), and circular plasmid (with one 1.5× telomere repeat) using *CmLEU2* marker transformed in C. albicans SN152: transformation efficiency, proportion of different types of transformants, and the number of autonomous transformants. Different types of transformants are represented by various shades of a color (lightest shade representing transients, intermediate shade representing ARS-transformants, and darkest shade representing integrants). The transformation efficiency and number of autonomous transformants are an average from three independent experiments. The size of circles showing proportion of transformant types is an approximation of transformation efficiency. (B) Mitotic stability (%) of ARS-transformants obtained with plasmids mentioned in panel A. The data represent the average from three independent ARS-transformants of each plasmid. Download FIG S4, PDF file, 0.2 MB. Copyright © 2019 Bijlani et al. 2019 Bijlani et al. This content is distributed under the terms of the Creative Commons Attribution 4.0 International license.

We next asked if linear plasmids carrying 1.5× TEL repeats were maintained autonomously. The ARS-transformants obtained exhibited moderate mitotic stability even after three passages, indicating that they were maintained autonomously over a few generations (Table S2). Southern blotting of DNA from a pLin-*CdARG4-ORI410* ARS-transformant with moderate mitotic stability (Fig. S2B) showed a single band with electrophoretic mobility similar to that of the naked linear DNA molecule used for transformation. We also recovered pLin-*CmLEU2*-*ORI410* molecules from ARS-transformants in E. coli (Fig. S5), demonstrating autonomous replication *in vivo*. Copy number of the linear plasmids in ARS-transformants, measured by qPCR, ranged widely (∼2 to 50 per cell, accounting for mitotic stability) (Fig. 2D).

10.1128/mSphere.00103-19.5 FIG S5 Steps for recovering pLin-*CmLEU2*-*ORI410* autonomously replicating plasmids from C. albicans ARS-transformants in E. coli. Three independent ARS-transformants were analyzed by this method. C, pCir-*CmLEU2*-*ORI410* plasmid control; M, marker. Download FIG S5, PDF file, 0.2 MB. Copyright © 2019 Bijlani et al. 2019 Bijlani et al. This content is distributed under the terms of the Creative Commons Attribution 4.0 International license.

10.1128/mSphere.00103-19.8 TABLE S2 Mitotic stability (averaged for three colonies) of ARS-transformants obtained with linear plasmids carrying *ORI410* after passaging. Download Table S2, PDF file, 0.1 MB. Copyright © 2019 Bijlani et al. 2019 Bijlani et al. This content is distributed under the terms of the Creative Commons Attribution 4.0 International license.

In contrast, *CaHIS1* transformants with moderate mitotic stability (45% and 63%) produced larger plasmid-hybridizing bands on Southern blots indicative of genomic integration (Fig. S2B). Further analysis of these *CaHIS1* integrants indicated gene replacement at the native locus by either double crossover or a gene conversion event (Fig. S3B). This is consistent with the idea that plasmids with a homologous marker can yield integrants apart from ARS-transformants.

### Effect of marker gene and *bona fide* ORIs on transformation parameters.

**FIG 3:**
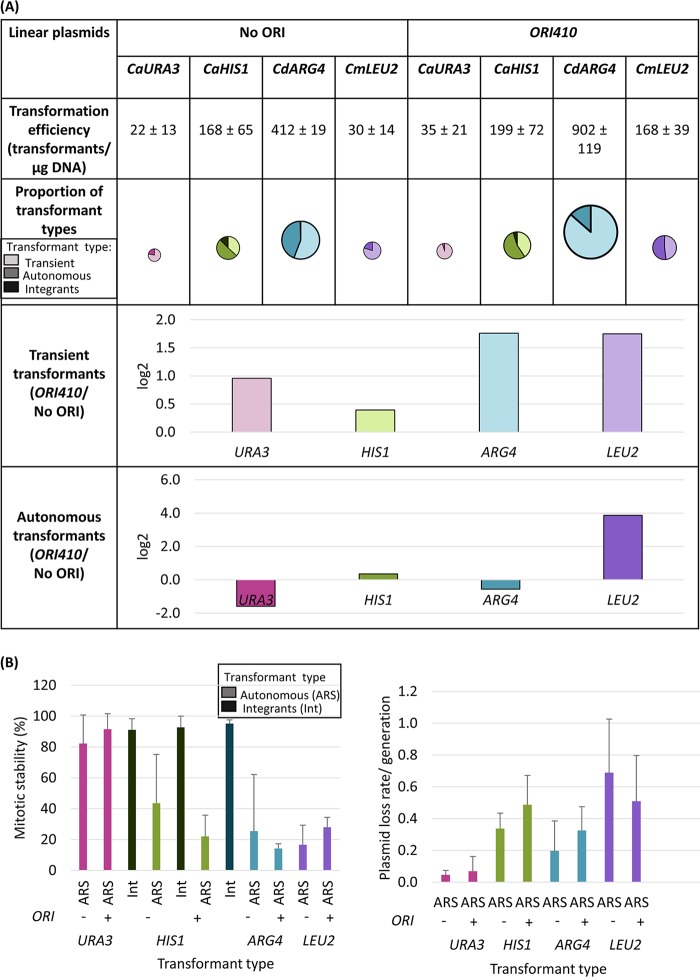
(A) Comparison of linear plasmids carrying different selection markers with and without *ORI410*: transformation efficiency, proportion of different types of transformants, and log_2_ value of the ratio of average number of transient or autonomous transformants with ORI to that without ORI (*ORI410*/No ORI). *CaURA3*, *CaHIS1*, *CdARG4*, and *CmLEU2* plasmids were transformed in C. albicans BWP17, SN76, SN76, and SN152, respectively. Different markers are represented by different colors, and different types of transformants are represented by various shades of a color (lightest shade representing transients, intermediate shade representing ARS-transformants, and darkest shade representing integrants). The transformation efficiency is an average from three independent experiments. The size of circles showing proportion of transformant types is an approximation of transformation efficiency. (B) Mitotic stability (%) of integrants and ARS-transformants and plasmid loss rate/generation for ARS-transformants obtained with different linear plasmids with and without *ORI410*. The data represent the average from three independent colonies of each type except for ARS-transformants with *CdARG4* and *CmLEU2* plasmids, where they represent the average from six independent colonies. Int, integrants; ARS, ARS-transformants.

We next asked to what extent a *bona fide* ORI sequence (*ORI410*) affected the transformation parameters of linear plasmids with different markers. With both homologous markers, *CaURA3* and *CaHIS1*, there were some integration events, initially more frequent for *CaHIS1*, although when propagated under selection, many of the *CaURA3* plasmids integrated (mitotic stability, ∼80 to 100%, and loss rate, <0.1 per generation; Fig. 3B). Furthermore, for *CaURA3* and *CdARG4,* addition of *ORI410* had no effect on the number of ARS-transformants (Fig. 3A) but improved plasmid stability compared to circular plasmids (Fig. 3B). This suggests that there may be a cryptic, intrinsic origin activity within *CaURA3* and *CdARG4* marker fragments (1.3 and 3.1 kb, respectively) that obviates the use of these markers to monitor the contribution of ORIs to plasmid replication and maintenance (discussed below). Similar results for *CaHIS1* and *CdARG4* were evident in different lab strains (Fig. S6). In contrast, addition of *ORI410* on pLin-*CmLEU2* resulted in an ∼5-fold increase in transformation efficiency, an ∼14-fold increase in ARS-transformants (Fig. 3A), and improved plasmid stability (Fig. 3B) relative to pLin-*CmLEU2* (*P* ≤ 0.005 and *P* ≤ 0.025 for total and ARS-transformants, respectively, using Student’s *t* test), suggesting that *CmLEU2* does not carry the intrinsic ARS activity seen on other markers.

10.1128/mSphere.00103-19.6 FIG S6 Comparison of pLin-*CaHIS1*-*ORI410* (A) and pLin-*CdARG4*-*ORI410* (B) transformation efficiency across different C. albicans strains. The transformation efficiency is an average from three independent experiments. Different types of transformants are represented by various shades of a color (lightest shade representing transients, intermediate shade representing ARS-transformants, and darkest shade representing integrants). Download FIG S6, PDF file, 0.6 MB. Copyright © 2019 Bijlani et al. 2019 Bijlani et al. This content is distributed under the terms of the Creative Commons Attribution 4.0 International license.

Given that all markers were inserted in the same position on a plasmid, which does not have any obvious origin-promoting sequence features, we tested the hypothesis that some feature required for origin firing is present at higher levels in *CaURA3*, *CaHIS1*, and *CdARG4* relative to *CmLEU2*, although many *CaHIS1* ARS-transformants integrate into the genome after additional passages. Since the lengths of *CaHIS1* and *CmLEU2* are similar, it seems unlikely that marker length is an important factor. Interestingly, the AT content of the two markers *CmLEU2* (62.3%) and *CaHIS1* (63.3%) with higher levels of ORI-dependent ARS-transformants (Fig. 3A) was below the average AT content of the C. albicans genome (66.7%), while the AT content of *CaURA3* (68.4%) and *CdARG4* (69.1%) was higher than that of the C. albicans genome. Thus, it appears that the markers with cryptic ORI function (*CaURA3* and *CdARG4*) that interferes with *bona fide* ORI activity have higher AT content. Of note, neither ORIs alone nor sequences on markers with possible cryptic ARSs share any obvious conserved primary sequence motifs. Based on its ORI dependence, *CmLEU2* is the most effective of the markers tested for comparing origin activity.

### Comparing different *bona fide* origins and *ORI410* fragments.

**FIG 4:**
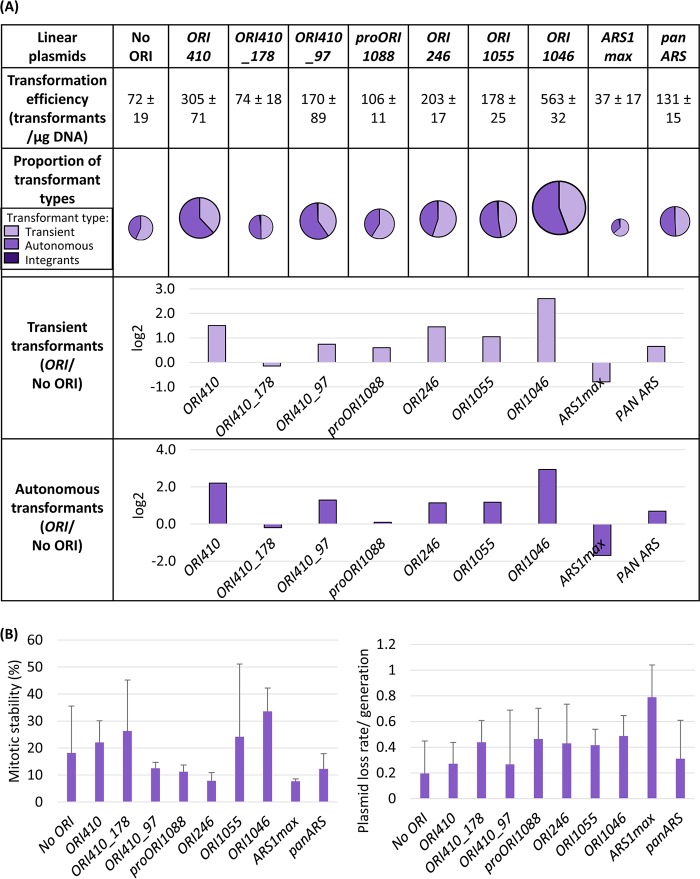
(A) Comparison of linear plasmids carrying *CmLEU2* marker with *ORI410* fragments, different *bona fide* ORIs, and heterologous ORIs transformed in C. albicans SN152: transformation efficiency, proportion of different types of transformants, and log_2_ value of the ratio of average number of transient or autonomous transformants with ORI to that without ORI (*ORI*/No ORI). Different types of transformants are represented by various shades of a color (lightest shade representing transients, intermediate shade representing ARS-transformants, and darkest shade representing integrants). The transformation efficiency is an average from three independent experiments. The size of circles showing proportion of transformant types is an approximation of transformation efficiency. (B) Mitotic stability (%) and plasmid loss rate/generation for ARS-transformants obtained with different linear plasmids mentioned in panel A. The data represent the average from six independent ARS-transformants of each plasmid.

Four ORIs from C. albicans (*ORI410* as well as *ORI1055*, *ORI1046*, and *ORI246*), defined previously as *bona fide* ORIs (28), were inserted into pLin-*CmLEU2* to examine their function compared to no-origin plasmids. All four *bona fide* ORIs yielded high transformation efficiencies (∼150 to 600/µg DNA) and ARS-transformants (∼75 to 300/µg DNA) (Fig. 4A) with moderate mitotic stability (10 to 45%) and plasmid loss rate (0.2 to 0.7 per generation) (Fig. 4B). *ORI1046* consistently yielded the highest transformation efficiency and ARS-transformants (∼300/µg DNA). Both negative-control plasmids, pLin-*CmLEU2* (No ORI) and that with pro*ORI1088,* a genomic ORC binding region that did not produce replication bubble arcs in 2-D gels (28), gave much lower transformation efficiency and ARS-transformants (31 and 44/µg DNA). Thus, all four *bona fide* ORIs can drive the origin-dependent autonomous replication of pLin-*CmLEU2* (Fig. 4A).

In S. cerevisiae, where the ACS is 11 bp, ARS function is detected only when the transforming fragment is ∼100 bp, including ACS (7, 8, 37, 38). We asked if two small overlapping fragments (178 bp and 97 bp) derived from *ORI410* (1.2 kb) (28) were able to retain minimum ARS function in C. albicans. *ORI410_97* had 2- to 3-times-higher transformation efficiency (∼170 transformants/µg DNA) and a ∼3-times-higher number of ARS-transformants than the no-ORI control (Fig. 4A). While the number of total and ARS-transformants for *ORI410_97* was lower than for the entire *ORI410*, the ARS-transformants had moderate mitotic stability (10 to 15%) and loss rate (∼0.4 per generation) (Fig. 4B). Thus, an ORI fragment of only ∼100 bp can drive linear plasmid replication and can yield ARS-transformants, which are 2- to 3-fold more stable than an analogous circular plasmid carrying the entire *ORI410* fragment.

### Heterologous ARSs.

C. albicans centromeres are regional and epigenetic, which contrasts with the point centromeres of S. cerevisiae (16). Since plasmid replication and origin function were difficult to demonstrate in C. albicans, we asked whether heterologous ARS fragments would function in C. albicans. The “*panARS*,” a 452-bp fragment from the K. lactis genome, functions as an active ORI in a range of *Saccharomycotina* yeast species with diverse ARS requirements, in some cases, even more efficiently than average homologous ARSs (e.g., Pichia pastoris) (31). The “*ARS1max*,” an origin from S. cerevisiae, was selected to drive better growth rates and lower plasmid loss rates than the original *ARS1* (39). Thus, we tested the ability of both the sequences to direct C. albicans plasmid replication on pLin-*CmLEU2* (pLin-*CmLEU2*+*panARS* and pLin-*CmLEU2*+*ARS1max*) relative to pLin-*CmLEU2*+*ORI410* and pLin-*CmLEU2*.

The pLin-*CmLEU2*+*panARS* plasmid resulted in ∼2- to 3-times-higher numbers of total and ARS-transformants relative to pLin-*CmLEU2* (Fig. 4A). The *panARS* ARS-transformants had moderate mitotic stability (∼10 to 20%) and loss rate (∼0.3 per generation) that were comparable to *ORI410_97* (Fig. 4B). This suggests that the sequence requirements of C. albicans origin function are at least partially conserved with those of K. lactis among other *Saccharomycotina* species. In contrast, pLin-*CmLEU2*+*ARS1max* had transformation parameters inferior to those of control plasmid pLin-*CmLEU2*: lower transformation efficiency, fewer ARS-transformants with the lowest mitotic stability (<8%), and the highest loss rate (∼0.8 per generation) (Fig. 4A and B) detected for any linear plasmid. This supports the idea that sequence requirements for origins in C. albicans (and other yeasts, for example, *Pichia pastoris* [40]) are distinct from those in S. cerevisiae.

## DISCUSSION

Early studies seeking potential origin sequences based on their ability to confer high transformation efficiency usually used circular plasmids with *CaURA3* as the selectable (and counterselectable) marker (22, 23, 25, 27). However, most transformants were highly unstable or rapidly integrated into the genome and thus were not useful for autonomous plasmid maintenance. Here we systematically compared four selectable markers in plasmids with circular or linear geometries to monitor the function of ORI sequences. Importantly, an ∼100-bp fragment (28) or the heterologous *panARS* (31) was sufficient to provide ARS function on a plasmid in C. albicans. This implies that sequence requirements for origin function in C. albicans are shared with distantly related yeasts. Nevertheless, the ability of cryptic ARSs on marker sequences to generate transient transformants implies that the sequence requirements for ARS function (and most likely chromosomal ORI function as well) are dependent on sequence context, possibly AT-richness, and other features that are not yet well understood.

An important insight from this work is that transformant colony size provides a useful preliminary indicator of plasmid mitotic stability. Presumably, colony size reflects the degree to which the plasmid replication and/or segregation enables growth of individual cells in a population under selective conditions. Specifically, in tiny or “pinpoint” colonies (25), plasmids are lost rapidly; in large colonies, plasmids are integrated stably (Table 1). In medium colonies, plasmids are moderately stable (Table 1) because they replicate autonomously, with some cells retaining the plasmid and others losing it. Of note, ARS-transformants with plasmids carrying homologous markers sometimes integrate in subsequent passages (generating larger colony subclones), a property less prevalent with the heterologous markers. Nonetheless, all markers on linear plasmids yield ARS-transformants, which initially can be identified based on colony size.

### Circular versus linear plasmids.

In S. cerevisiae, linear plasmids and minichromosomes were used to study chromosome components and to propagate large segments of DNA (41, 42). However, most work was done with circular plasmids that are readily propagated in E. coli; propagation of linearizable plasmids with inverted telomere repeats (29, 43–45) was labor-intensive and subject to recombination of the repeats in E. coli. Here, a simple approach obviates many of these technical challenges by synthesizing linear plasmids from circles immediately prior to transformation (Fig. 2A). Thus, the two plasmid geometries are directly comparable, differing only by the presence or absence of 1.5× TEL repeats.

Does the presence of TEL sequence alone improve the segregation of linear versus circular plasmids? In C. albicans as in S. cerevisiae, adding TEL sequences to linear plasmids improves their stability (46) (Fig. 2C). However, adding TEL sequences does not improve circular plasmid segregation in C. albicans (see Fig. S4 in the supplemental material); by contrast, TEL sequences on circles stabilized *ScARS* plasmids (47) and antagonized the segregation of *ScCEN* plasmids (48). Thus, *Ca*TEL sequence function is required for autonomous linear plasmid maintenance and is dependent upon its geometry: in a chromosome end context, but not within a circular context. This supports the idea that interactions between nonterminal TEL DNA and telomeric proteins likely differ between C. albicans and S. cerevisiae and that linear plasmids require telomere ends to remain stable.

In S. cerevisiae, noncentromeric plasmids are retained in the mother cells due to their attachment to the nuclear membrane (49) as well the presence of a diffusion barrier at the bud neck (50). It is tempting to speculate if this is also true for circular plasmids (with or without TEL) in C. albicans. Whether and how the linear plasmids might be better able to segregate to daughter cells remain to be explored.

### Effect of selectable markers.

Comparison of the markers found that *CaURA3* was not ideal, which explains difficulties in many earlier investigations (25, 26), and addition of *LEU2* or *HIS1* to *CaURA3* plasmids relied on *URA3* selection as well (22, 27). Studies selecting for *IMH3^R^* or *CaADE2* found that putative ARS-transformants integrated at high frequency (21, 25). Sometimes integration events involved and/or altered the putative origin structure (24), and the resulting plasmids were not maintained autonomously. Notably, *CaURA3* linear plasmid produced very few ARS-transformants, with or without ORI addition, and these eventually integrated into the genome (Fig. 3). In contrast, ARS-transformants with either *CaHIS1*, *CdARG4*, or *CmLEU2* were maintained over three passages (Table S2).

We suggest the appearance of transient transformants cannot be used to define origin function on a plasmid, especially when the *CaURA3* marker with latent origin activity is used. Therefore, transients seen with *ORI7-L1* and *-R1* cannot be used to draw conclusions about the function of these chromosomal regions as origins, especially since the published data lack a control plasmid containing the *CaURA3* marker without an origin (26). Transient transformants with these origins have been used to postulate that centromere function required a preexisting origin. However, our results showing that these chromosomal regions do not act as origins, together with published neocentromere locations at chromosomal regions that did not contain preexisting origins (51, 52), support a model where kinetochore assembly can convert a nonorigin region to an origin. Furthermore, if many genome sequences can recruit replication factors and provide weak origin function on a plasmid as in S. pombe (14, 53), it is not surprising that sequences within neocentromere regions may recruit origins to new loci. The dramatically increased origin efficiency of the neocentromeric loci is likely due to neocentromere-mediated recruitment of replication initiation activities like Cdc7-Dbf4, which is normally found at wild-type centromeres (54).

Heterologous *CdARG4* did not integrate frequently, yet, it gave high numbers of ARS-transformants in the absence of an added ORI. We posit that both *CaURA3* and *CdARG4* have weak intrinsic ARS activity and that this may compete with a *bona fide* ORI when both are on a plasmid. This suggests that C. albicans, like S. pombe, has “cryptic origins” (55), i.e., sites that are normally not used for replication initiation yet have the potential to form active replication origins. It also suggests that, once a cryptic origin has been established, it can continue to function, perhaps because, once well established in an ARS-transformant, a weak origin may be more likely to fire in the next cell cycle. The requirements for cryptic ARS function remain elusive. We cannot rule out the possibility that chromatin structure and topological constraints might affect ARS activity.

Why might inefficient ORIs interfere with *bona fide* ORI activity? In S. cerevisiae, two ORIs in close proximity in the genome interfere with each other (56). Three mechanisms were proposed to explain this: (i) timing of ORI firing might differ such that the nonfiring ORI is replicated passively, (ii) DNA at the two ORIs might interfere topologically (e.g., via altered supercoiling), or (iii) the two origins may compete for a limited number of licensing factors (e.g., ORC-associated proteins) (56). Interestingly, the orientation of ORC sites relative to one another could also be relevant (57), and all six predicted ORC sites (28) on *CmLEU2* are oriented in the same direction, while predicted ORC sites (28) on the other three markers were found in both orientations. While mechanisms of *Ca*ORI and *Sc*ORI firing are likely to differ to some degree, these options may explain the phenomenon in C. albicans as well.

Most organisms do not have highly defined ARS consensus sequences, and it appears that this is the case in C. albicans as well. In S. pombe, ORIs have average AT contents ranging from 72 to 75% (58), with an average of 64% in the genome. *CaURA3* and *CdARG4* have 68.4% and 69.1%, respectively, with an average of 66.7% AT content in the genome. Furthermore, for all four markers on linear plasmids, the number of poly(A) tracts (≥3 nucleotides, normalized for marker length) correlated well (*R*^2^ = 0.85) with the number of transients obtained. This is consistent with the idea that AT-rich sequences and/or poly(A) tracts may attract replication factors and acquire cryptic ORI function. This, in turn, might interfere with *bona fide* ORI firing on the plasmid by mechanisms like those proposed for S. cerevisiae (56).

### Testing origin function.

The linear *CmLEU2* plasmid backbone provided the first opportunity to compare the efficiencies of different *bona fide* ORIs, *ORI410*-derived fragments (28), and heterologous origins (31, 39). All four *bona fide* ORIs yielded high numbers of ARS-transformants as well as moderate mitotic stability and loss rates (Fig. 4). We do not know why the 178-bp fragment, *ORI410_178*, had little or no obvious origin function while a smaller fragment derived from it (*ORI410_97*) was active. Clearly, DNA primary sequence is not sufficient to confer ORI function. We presume that sequence features together with their context relative to other plasmid components affect ORI activity, which has been seen on *Sc*ARS plasmids as well (59). Importantly, the synthetic *panARS*, which was derived from K. lactis (31), had transformation parameters similar to those of *ORI410_97*. Thus, the requirements for replication origin function in C. albicans are at least partially conserved with other *Saccharomycotina* species, and *panARS* provides a heterologous ORI that should not integrate into the genome. We suggest that it might be possible to whittle down the 452-bp *panARS* to generate a relatively good heterologous ORI of ∼100 bp.

### Summary.

ARS function can be studied in C. albicans using a heterologous marker and a *bona fide* ORI of as small as ∼100 bp or the heterologous *panARS* on linear plasmids carrying 1.5× TEL ends. Importantly, linear plasmid conformation greatly facilitates transformation efficiency and mitotic stability. Unexpectedly, the choice of selectable marker has a major effect on the degree to which plasmids are maintained autonomously. To date, *CmLEU2* is the single marker that has a low level of intrinsic cryptic origin activity and rare integration events, making it ideal for studying origin activity on a plasmid. The linear plasmids described here fill a major gap in the tools available for conventional molecular manipulations of C. albicans and will facilitate our ability to study molecular aspects of ORI, telomeric, and centromeric structure and function.

## MATERIALS AND METHODS

### Strains, plasmids, primers, and growth conditions.

Yeast strains and plasmids used are listed in Tables 2 and 3, respectively. Primers used are provided in Table 4.

**TABLE 2:** List of strains used in the study

Strain no.	C. albicans strain	Genotype	Reference	Gene(s) used with
YJB-T 45	BWP17	*ura3*::λ*imm^434^/ura3*::λ*imm^434^ his1*::*hisG/his1*::*hisG arg4*::*hisG/arg4*::*hisG*	65	*CaURA3*
YJB-T 72	SN76	*ura3-iro1*::*imm^434^*/*ura3-iro1*::*imm^434^ his1*::*hisG*/*his1*::*hisG arg4*Δ/*arg4*Δ	30	*CaHIS1*, *CdARG4*
YJB-T 736	SN152	*ura3-iro1*::*imm^434^*/*URA3-IRO1 his1*::*hisG*/*his1*::*hisG arg4*Δ/*arg4*Δ *leu2*Δ*/leu2*Δ	66	*CmLEU2*
YJB-T 65	SN95	*ura3-iro1*::*imm^434^*/*URA3-IRO1 his1*::*hisG*/*his1*::*hisG arg4*Δ/*arg4*Δ	30	*CaHIS1*, *CdARG4*

**TABLE 3:** List of plasmids used in the study

Plasmid no.	Description	Reference or source
BJB-T1	pGEM-*URA3*	65
BJB-T226	pGEM-*URA3*-*ORI410*	This study
BJB-T2	pGEM-*HIS1*	65
BJB-T140	pGEM-*HIS1-ORI410*	This study
BJB-T391	pGEM-*CdARG4*	This study
BJB-T234	pGEM-*CdARG4-ORI410*	This study
BJB-T230	pGEM-*CmLEU2*	This study
BJB-T231	pGEM-*CmLEU2-ORI410*	This study
BJB-T398	pGEM-*CmLEU2-ORI410_178*	This study
BJB-T399	pGEM-*CmLEU2-ORI410_97*	This study
BJB-T400	pGEM-*CmLEU2-proORI1088*	This study
BJB-T401	pGEM-*CmLEU2-ORI246*	This study
BJB-T402	pGEM-*CmLEU2-ORI1055*	This study
BJB-T403	pGEM-*CmLEU2-ORI1046*	This study
BJB-T404	pGEM-*CmLEU2-ARS1max*	This study
BJB-T405	pGEM-*CmLEU2-panARS*	This study
BJB-T227	pGEM-*URA3*-*ORI7-R1*	This study
BJB-T228	pGEM-*URA3*-*ORI7-L1*	This study

**TABLE 4:** List of primers used in the study

Primer no.	Primer sequence (5′–3′)^*a*^	Purpose
BP196	aggcaatagcatttccatctggtttcttgtcgaccatatgGGAACATCTGAAATTGGTTC	Primer to amplify *ORI410* to clone in pGEM-*CaHIS1*
BP197	gaatactcaagctatgcatccaacgcgttgggagctctccTTGATGATTGGATCGGGTTC	Primer to amplify *ORI410* to clone in pGEM-*CaHIS1*
BP1266	gcatgctcccggccgccatggccgcgggatGTAACGGCCGCCAGTGTG	Primer to amplify *CdARG4* and *CmLEU2* for cloning in pGEM and pGEM-*ORI410*
BP1267	catccaacgcgttgggagctctcccatatgCCAGTGTGATGGATATCTGCAG	Primer to amplify *CdARG4* and *CmLEU2* for cloning in pGEM and pGEM-*ORI410*
BP1262	CATATGGGAGAGCTCCCAACGCGTTG	Forward primer to amplify *pGEM* backbone from pGEM-*CaHIS1* to clone *CdARG4* and *CmLEU2*
BP1265	CATATGGGAACATCTGAAATTGGTTCTTTGGTAGATCTGCC	Forward primer to amplify *pGEM-ORI410* backbone from pGEM-*CaHIS1*-*ORI410* to clone *CdARG4* and *CmLEU2*
BP1263	ATCCCGCGGCCATGGCGG	Reverse primer to amplify *pGEM* backbone from pGEM-*CaHIS1* and pGEM-*CaHIS1*-*ORI410* to clone *CdARG4* and *CmLEU2*
BP1246	GTCGACCTGCAGGCGGCC	Primer to amplify pGEM-*CaURA3* to clone ORI
BP1247	GGAGAGCTCCCAACGCGTTG	Primer to amplify pGEM-*CaURA3* to clone ORI
BP1248	aatcactagtgcggccgcctgcaggtcgacTTGTAGATTTCAAAAATGCTTC	Primer to clone *ORI7-L1* in pGEM-*CaURA3*
BP1249	gctatgcatccaacgcgttgggagctctccGATTTGTGTGTGCTTACTAGAG	Primer to clone *ORI7-L1* in pGEM-*CaURA3*
BP1250	aatcactagtgcggccgcctgcaggtcgacTTGTGTAGTAAAGGGTTGTTG	Primer to clone *ORI7-R1* in pGEM-*CaURA3*
BP1251	gctatgcatccaacgcgttgggagctctccAGTTAGGAAGAGTATAAATATGTGTAGTC	Primer to clone *ORI7-R1* in pGEM-*CaURA3*
BP1198	ttctgcagatatccatcacactggcatatgACAAAAAATCATTAGCAAAATATTC	Primer to amplify *ORI410_178* to clone in pGEM-*CmLEU2*
BP1199	gctatgcatccaacgcgttgggagctctccCCAGTGGAATTTGCAACC	Primer to amplify *ORI410_178* to clone in pGEM-*CmLEU2*
BP1200	ttctgcagatatccatcacactggcatatgACTTTCAGAAATTGGTTGG	Primer to amplify *ORI410_97* to clone in pGEM-*CmLEU2*
BP1201	gctatgcatccaacgcgttgggagctctccACACAAAAAATCATTAGCAAAATATTC	Primer to amplify *ORI410_97* to clone in pGEM-*CmLEU2*
BP1214	ttctgcagatatccatcacactggcatatgAGCAGTTTTAAAATAAATAGGG	Primer to amplify *proORI1088* to clone in pGEM-*CmLEU2*
BP1215	gctatgcatccaacgcgttgggagctctccTTGGATTATCAAAAAATCATTAG	Primer to amplify *proORI1088* to clone in pGEM-*CmLEU2*
BP1194	ttctgcagatatccatcacactggcatatgTGTTGCAAAATATGAGTAAAAAAA	Primer to amplify *ORI246* to clone in pGEM-*CmLEU2*
BP1195	gctatgcatccaacgcgttgggagctctccACAACGGAGGGTAAGGTG	Primer to amplify *ORI246* to clone in pGEM-*CmLEU2*
BP1192	ttctgcagatatccatcacactggcatatgTGGTTATGTACTTGATCACCC	Primer to amplify *ORI1055* to clone in pGEM-*CmLEU2*
BP1193	gctatgcatccaacgcgttgggagctctccTACAGAATGAGTAATATACAATGTTTG	Primer to amplify *ORI1055* to clone in pGEM-*CmLEU2*
BP1196	ttctgcagatatccatcacactggcatatgATATATTTGTGATTCAACCACAC	Primer to amplify *ORI1046* to clone in pGEM-*CmLEU2*
BP1197	gctatgcatccaacgcgttgggagctctccCAAAAATATCTCGTGAATCTTTTC	Primer to amplify *ORI1046* to clone in pGEM-*CmLEU2*
BP1186	ttctgcagatatccatcacactggcatatgCACATGTTAAAATAGTGAAGGAG	Primer to amplify *ARS1max* to clone in pGEM-*CmLEU2*
BP1187	gctatgcatccaacgcgttgggagctctccAAAGCTTACATTTTATGTTAGCTG	Primer to amplify *ARS1max* to clone in pGEM-*CmLEU2*
BP1188	ttctgcagatatccatcacactggcatatgTCAACATCTTTGGATAATATCAG	Primer to amplify *panARS* to clone in pGEM-*CmLEU2*
BP1189	gctatgcatccaacgcgttgggagctctccTAGTGCTGATTATGATTTGACG	Primer to amplify *panARS* to clone in pGEM-*CmLEU2*
BP1179	CATATGCCAGTGTGATGGATATCTG	Primer to amplify pGEM-*CmLEU2* to clone ORIs
BP1180	GGAGAGCTCCCAACGCGT	Primer to amplify pGEM-*CmLEU2* to clone ORIs
BP1204	ACTGGCCGTCGTTTTACA	Primer to amplify linear plasmids without TEL
BP1205	GAATTGTAATACGACTCACTATAGGG	Primer to amplify linear plasmids without TEL
BP1252	CCGTACACCAAGAAGTTAGACATCCGTACACCAActtaagggatccgcatgctcccggccgccatg	Primer to amplify linear plasmids with 1.5× TEL repeat
BP1253	CCGTACACCAAGAAGTTAGACATCCGTACACCAActtaagggatccgggcccaattcgccctatag	Primer to amplify linear plasmids with 1.5× TEL repeat
BP1254	CCGTACACCAAGAAGTTAGACATCCGTACACCAAGAAGTTAGACATCCGTACACCAA cttaagggatccgcatgctcccggccgccatg	Primer to amplify linear plasmids with 2.5× TEL repeats
BP1255	CCGTACACCAAGAAGTTAGACATCCGTACACCAAGAAGTTAGACATCCGTACACCAA cttaagggatccgggcccaattcgccctatag	Primer to amplify linear plasmids with 2.5× TEL repeats
BP1843	CAAGGCGAGTTACATGATCC	Primer to amplify *AMP^R^* for qPCR
BP1844	GGATGGCATGACAGTAAGAG	Primer to amplify *AMP^R^* for qPCR
BP285	TTTGTACTTAGCGGCTACCTG	Primer to amplify chromosome 1L *CEN* for qPCR
BP317	GAAAGAAGTGGGAGGAAAGGG	Primer to amplify chromosome 1L *CEN* for qPCR
BP1869	CATGTATGGTAATCCAAATGGG	Forward primer that anneals outside 5′ UTR of *CaHIS1*
BP1870	AACACGGTGCACCAGTC	Reverse primer that anneals outside 3′ UTR of *CaHIS1*
BP1841	GGCTGGCTGGTTTATTGC	Reverse primer that anneals to *AMP^R^* gene
BP1873	GGTAATGTAATGGACGAATTGAAG	Forward primer that anneals within *CaHIS1*
BP1857	CAACCTGGGTATTGATATGTTG	Reverse primer that anneals to *CmLEU2* promoter

a Sequences in lowercase indicate regions homologous to the plasmid.

C. albicans strains were grown at 30°C in YPAD medium (60) or SD minimal medium or SD-Complete medium (60) containing leucine at 170 mg/liter and all other amino acids (Sigma-Aldrich, USA) at 85 mg/liter.

E. coli DH5α was used for all cloning experiments and was grown in LB medium (60) at 37°C with ampicillin (Sigma-Aldrich, USA) at 100 µg/ml.

### Cloning of selection markers and ORIs in plasmids.

Selection markers and ORIs were amplified with primers (Table 4) carrying 15- to 40-bp homology to the vector and ∼20-bp homology to the marker or ORI fragment. Amplified vector and insert (1:3 ratio) were assembled in 20 µl Gibson reaction mixture (61) per the manufacturer’s instructions (New England BioLabs [NEB], USA), and 2 µl was transformed into chemically competent E. coli (NEB, USA). Following selection on LB plus ampicillin overnight, recombinants were detected by colony PCR using primers to the vector, outside the cloning sites. Putative positive clones were then confirmed by Sanger sequencing.

### Construction of linear plasmids.

Linearizing primers (BP1252, BP1253, BP1254, and BP1255; Table 4) contained (from 5′ and 3′) a 34- or 57-nt telomere sequence (36), AflII and BamHI recognition sites, and then homology to the plasmid AatII site. Linear plasmids were amplified from circular plasmids (Fig. 2A) by two-step PCR using Kappa HiFi HotStart polymerase (Roche, Switzerland). Cycling conditions were as follows: 98°C denaturation step for 30 s, 30 cycles of 98°C (10 s) and 72°C (30 s/kb), and final extension of 72°C for 10 min.

To generate linear plasmids without telomeric ends, the circular plasmids were amplified with primers (BP1204 and BP1205; Table 4) using Phusion polymerase (Thermo Fisher Scientific, USA). Cycling conditions were as follows: 98°C denaturation step for 30 s; 25 cycles of 98°C (10 s), 60°C (30 s), and 72°C for (30 s/kb); and a final extension step at 72°C for 10 min.

### Colony PCR.

A small portion of the colony was resuspended in the PCR with *Taq* polymerase (Hy-*Taq* Ready Mix, Hy-labs, Israel). Cycling conditions were as follows: 95°C denaturation step for 5 min; 25 cycles of 95°C (30 s), annealing at a primer-dependent temperature (30 s), and 72°C (1 min/kb); and a final extension step at 72°C for 5 min.

### High-efficiency transformation of C. albicans.

C. albicans transformation was carried out as described previously (62) with the only difference being that DTT was added at a final concentration of 25 mM and that after a 45-min incubation with lithium acetate (LiAc)-TE, the cells were further incubated with DTT for 1.5 h.

### Mitotic stability assay.

Yeast transformants were inoculated into SDC(−AA) (selective) medium and grown overnight at 30°C. The cultures were 10-fold serially diluted, and an appropriate dilution to yield 100 to 200 colonies was plated onto both SDC(−AA) and SDC plates. The plates were incubated at 30°C, and the number of colonies was counted after 2 days. Mitotic stability was calculated as [no. of colonies on SDC(−AA)/no. of colonies on SDC] × 100.

### Plasmid loss assay.

Yeast transformants grown overnight in SDC(−AA) for mitotic stability assay were diluted 100-fold into SDC medium and grown overnight at 30°C. The cultures were 10-fold serially diluted, and 5 µl of each dilution was spotted on both SDC(−AA) and SDC plates. The plates were incubated at 30°C for 2 days, and the number of colonies was counted from the highest dilution at which they were well separated. The proportion of cells that retained the plasmid without selection was calculated as [no. of colonies on SDC(−AA)/no. of colonies on SDC] from the same dilution. The plasmid loss rate was then determined as described in the work of Longtine et al. (47).

### Southern blotting.

The genomic DNA was extracted from 10-ml overnight-grown cultures in SDC(−AA) as described previously (62). Fifteen to 20 µg genomic DNA was digested overnight with ApaI and run on a 1% agarose gel for 16 to 20 h at 1.4 V/cm. Southern blotting was performed as described previously (63). A PCR fragment of the *AMP^R^* gene was used to probe the plasmids on the blot.

### qPCR to determine plasmid copy number.

qPCR was carried out with the genomic DNA from autonomous transformants using SYBR green master mix (Bio-Rad, USA) per the manufacturer’s protocol in a Bio-Rad CFX96 Touch real-time PCR detection system. Cycling conditions were as follows: 95°C (3 min), 40 cycles of 95°C (5 s) and 60°C (30 s), and melt curve from 65.0 to 95.0 for 5 s. The *AMP^R^* gene was used to determine plasmid copy number, and *CEN* of chromosome 1 was used as a reference gene. The two primer sets used had similar efficiencies in the reaction; therefore, fold change in the copy number of plasmids was determined relative to the genomic control. Copy number of plasmids was calculated as copy number = (fold change × 2)/mitotic stability.

### Growth rate determination.

From fresh transformation plate, three independent colonies per colony size were inoculated into 2 ml SDC(−AA) and grown overnight at 30°C and 250 rpm. Fifty microliters of cell culture was washed with ddH_2_O and resuspended in 1 ml SDC(−AA); 10 µl was inoculated in 100 µl SDC(−AA) in a 96-well round-bottom sterile polystyrene plate (Corning). For tiny colonies that could not be propagated in liquid medium, three independent colonies were directly inoculated from the plate into 100 µl SDC(−AA). The plate was subsequently incubated at 30°C in a Tecan Infinite F200 Pro (Tecan, Switzerland) microplate incubator/spectrometer with a shaking duration of 900 s, and the OD_600_ was collected every 15 min over a 24-h period. OD versus time was plotted to generate growth curves.

### Recovery of pLin-*CmLEU2*-*ORI410* plasmid from autonomous transformants.

The genomic DNA from the yeast transformants was digested with BamHI (NEB, USA) to cut the linear plasmid at both of the ends, resulting in removal of telomere repeats. The digested DNA was ligated overnight with T4 DNA ligase (Thermo Fisher Scientific, USA). The ligation product was transformed into electrocompetent E. coli (64), and the clones obtained were confirmed by PCR primers flanking the ligation site followed by sequencing.
