# Supplementary figures and images for: Autonomously Replicating Linear Plasmids That Facilitate the Analysis of Replication Origin Function in Candida albicans
Source: mSphere. 2019 Mar 6;4(2):e00103-19. doi: 10.1128/mSphere.00103-19 (PMC6403455; doi:10.1128/mSphere.00103-19)

Figure S1

(A)

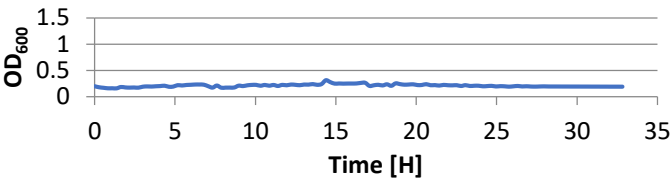

(B)

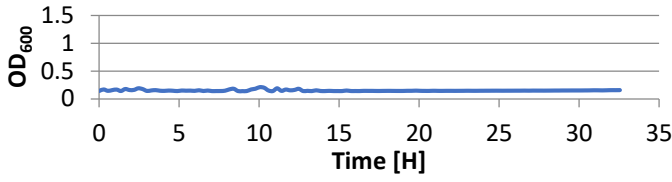

(C)

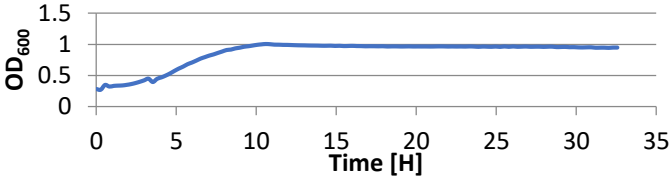

(D)

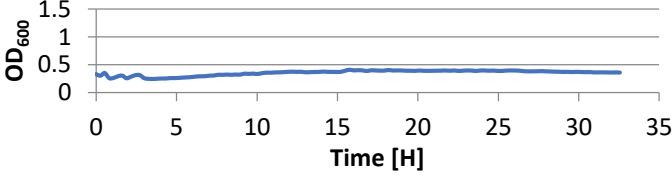

(E)

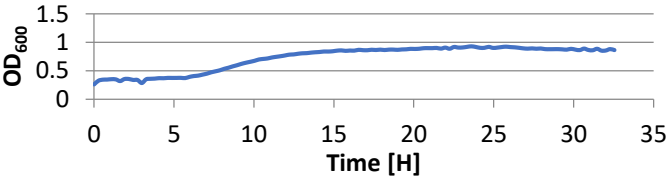

(F)

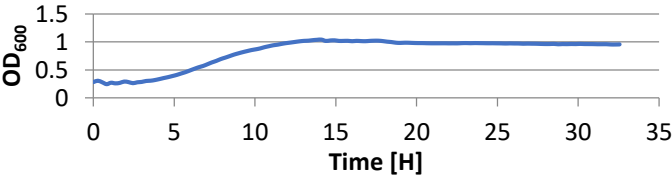

Supplement: FIG S1 [file mSphere.00103-19-sf001.pdf]

Figure S2

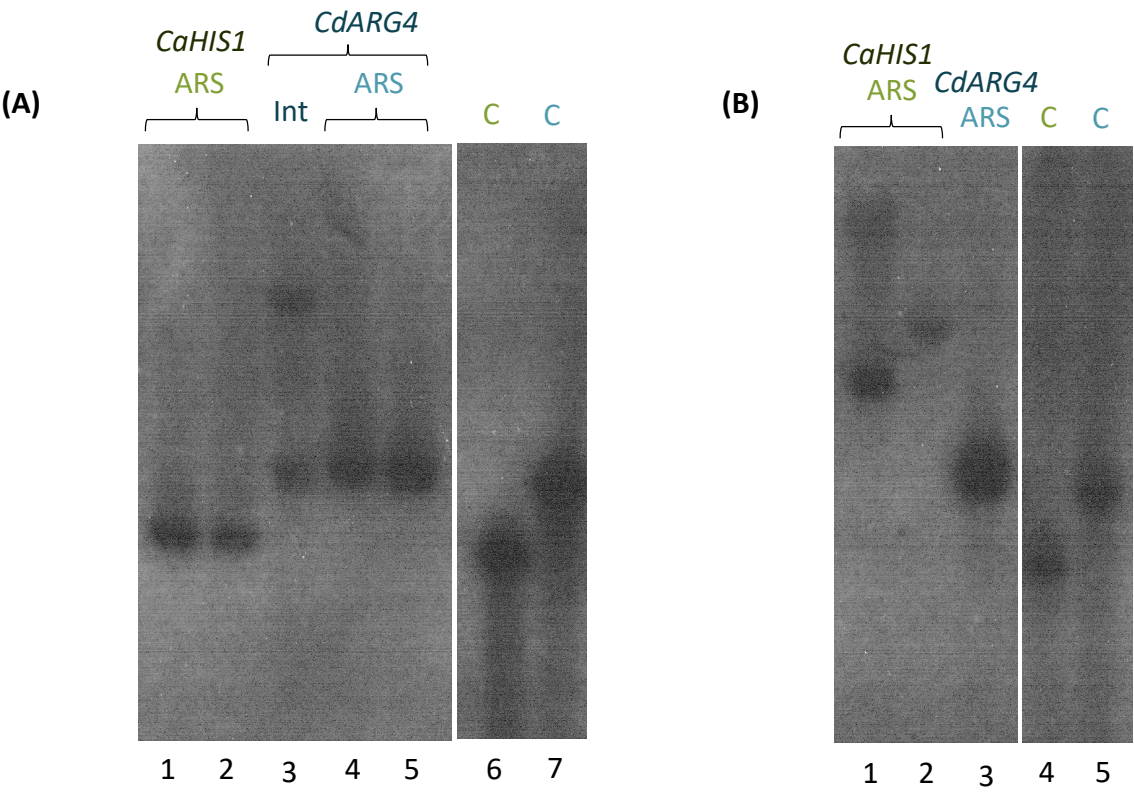

Supplement: FIG S2 [file mSphere.00103-19-sf002.pdf]

Figure S4

(A)

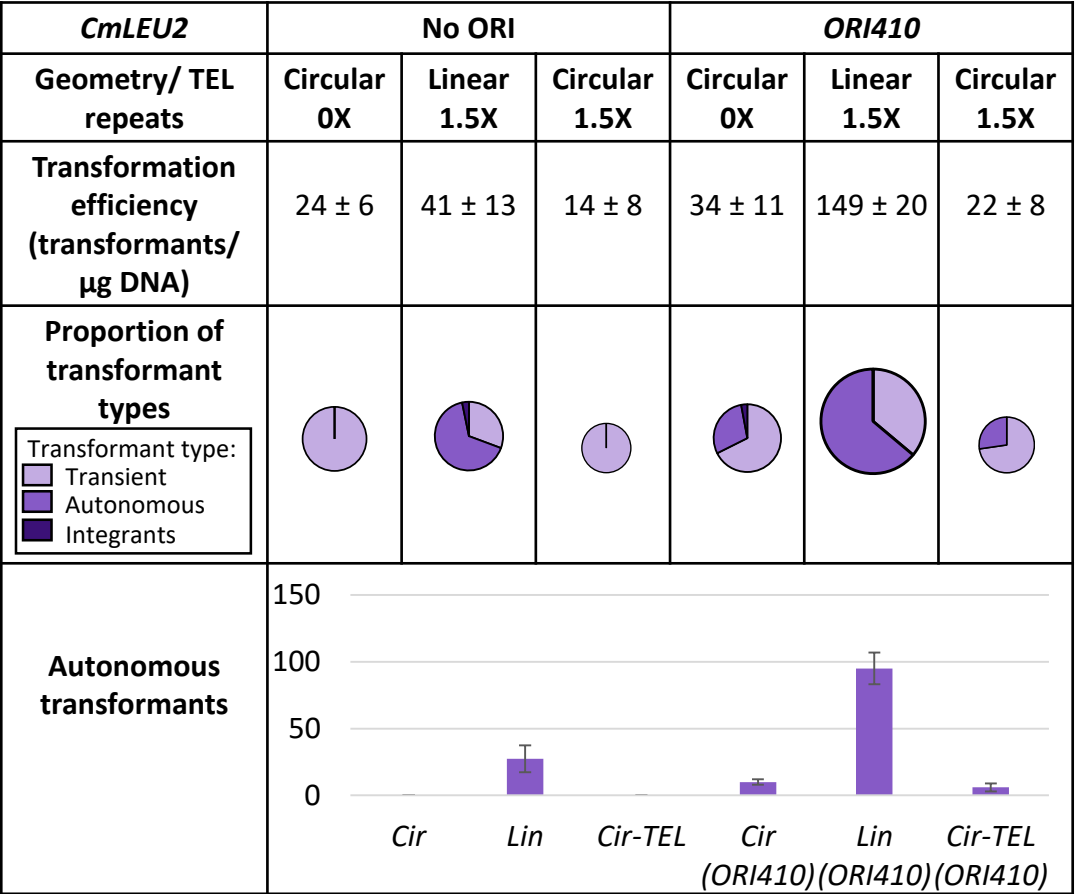

(B)

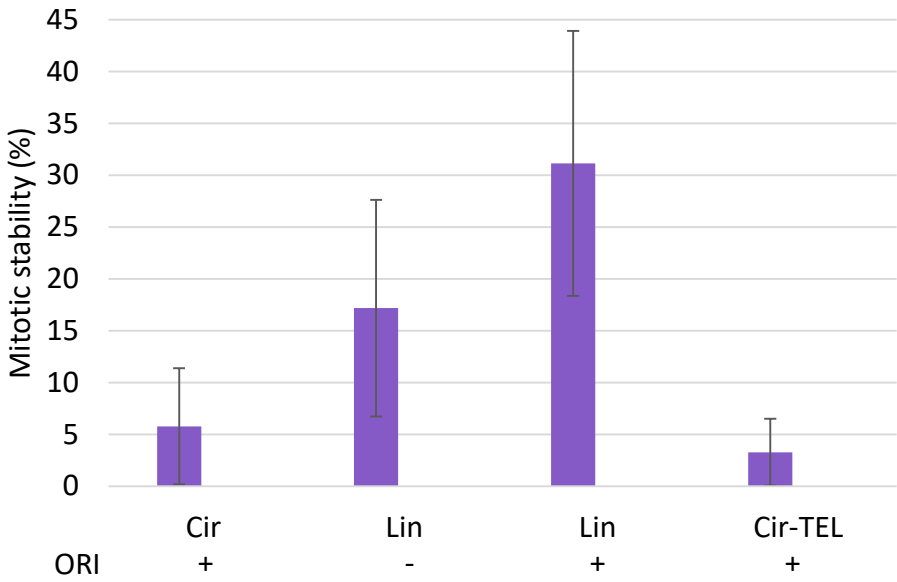

Supplement: FIG S4 [file mSphere.00103-19-sf004.pdf]

Figure S5

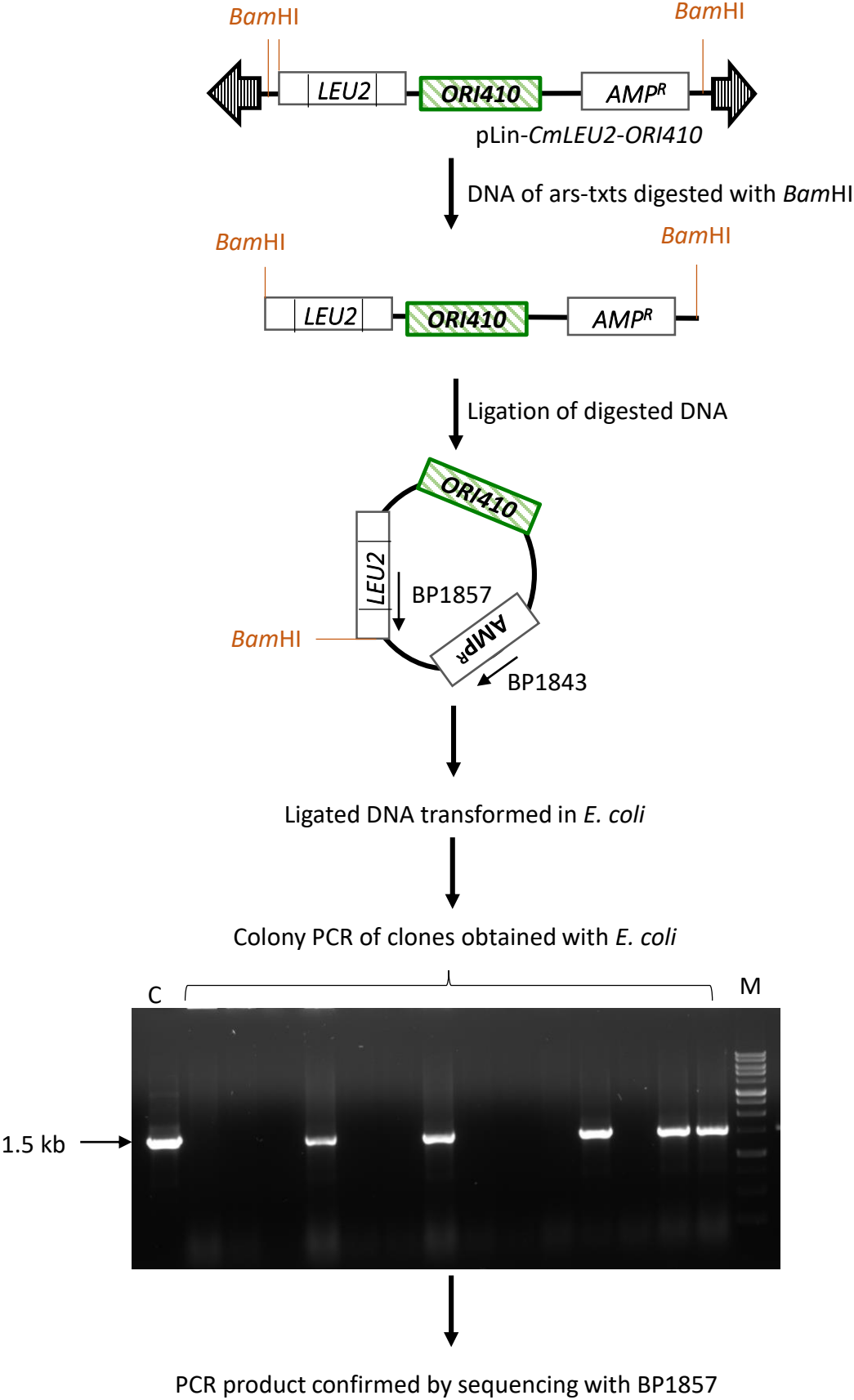

Supplement: FIG S5 [file mSphere.00103-19-sf005.pdf]

Figure S6 (A)

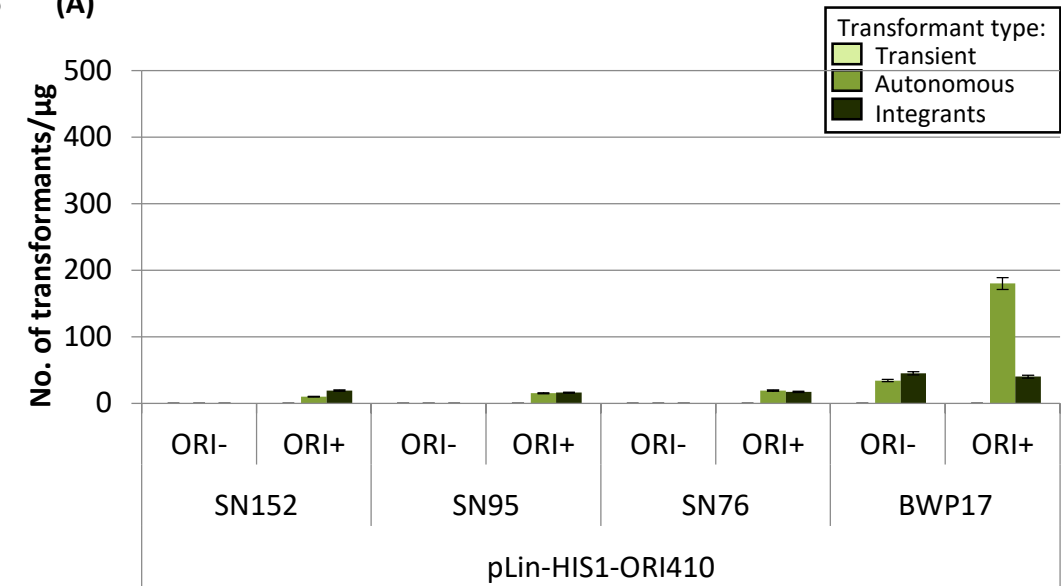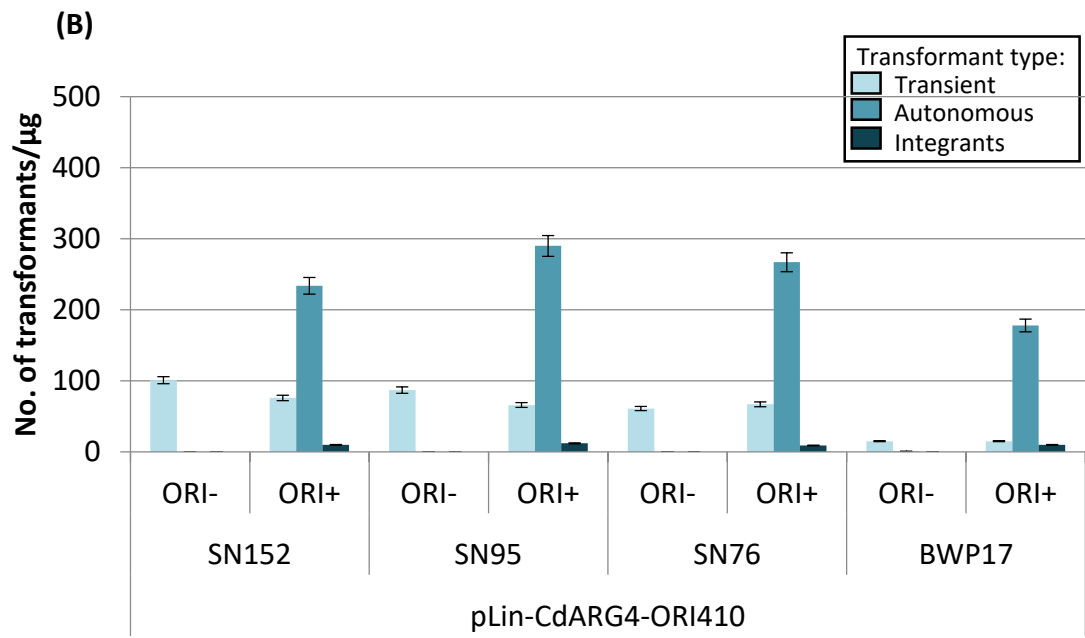

Supplement: FIG S6 [file mSphere.00103-19-sf006.pdf]
