# Supplementary material for: Autonomously Replicating Linear Plasmids That Facilitate the Analysis of Replication Origin Function in Candida albicans
Source: mSphere. 2019 Mar 6;4(2):e00103-19. doi: 10.1128/mSphere.00103-19 (PMC6403455; doi:10.1128/mSphere.00103-19)
Supplement: TABLE S1 [file mSphere.00103-19-st001.pdf]

Table S1.

**Strain**	**Origin**	**Transformation efficiency (pCir-CaURA3)**
		**Tiny**
**BWP17**	No ORI	20
	ORI410	79
	ORI7-R1	2
	ORI7-L1	8
	No DNA	0
**SN76**	No ORI	3
	ORI410	4
	ORI7-R1	0
	ORI7-L1	5
	No DNA	0
