# Supplementary material for: Autonomously Replicating Linear Plasmids That Facilitate the Analysis of Replication Origin Function in Candida albicans
Source: mSphere. 2019 Mar 6;4(2):e00103-19. doi: 10.1128/mSphere.00103-19 (PMC6403455; doi:10.1128/mSphere.00103-19)

Figure S3 (A) Integration of circular plasmid

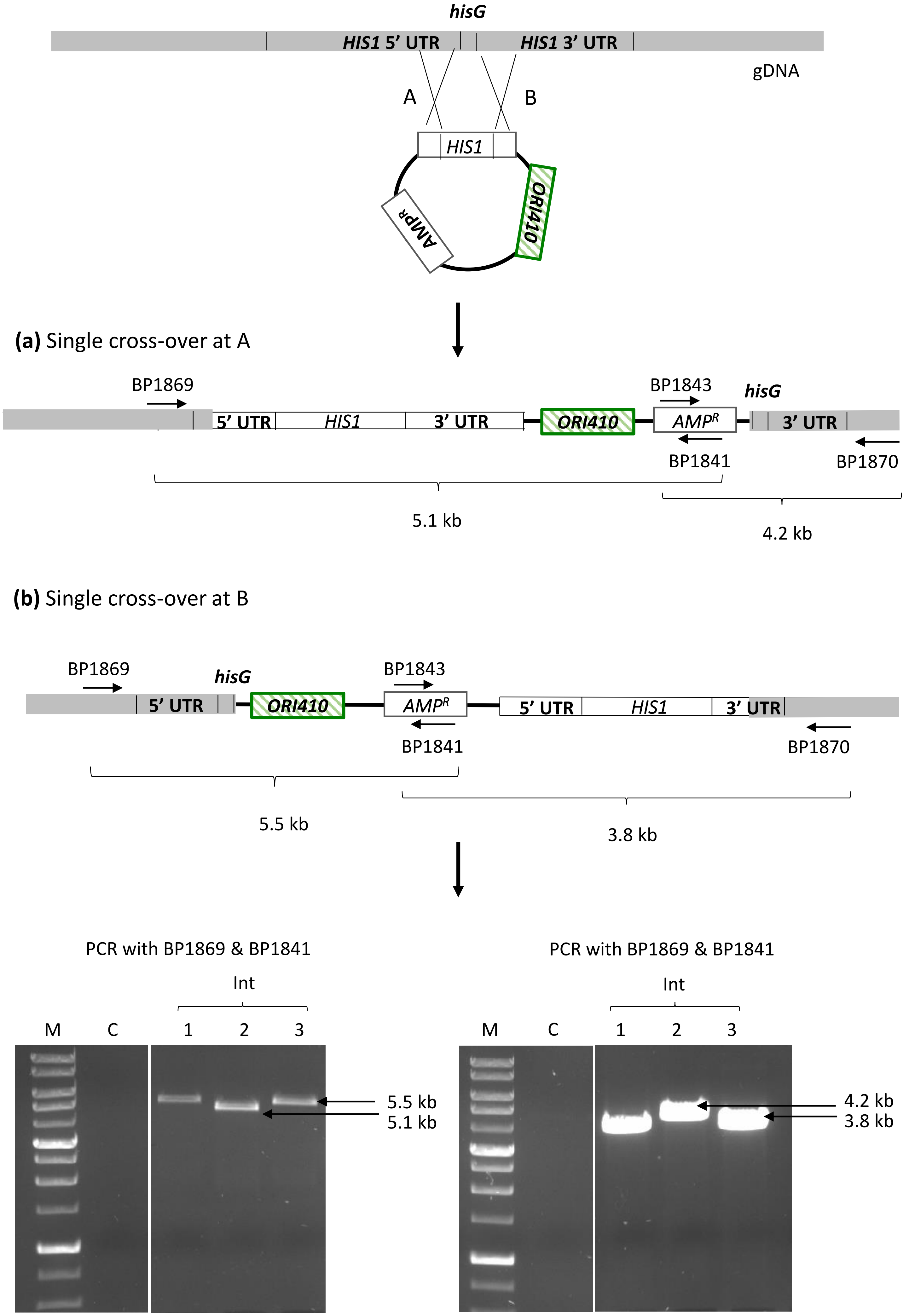

Figure S3 **(B) Integration of linear plasmid by double cross-over at *HIS1***

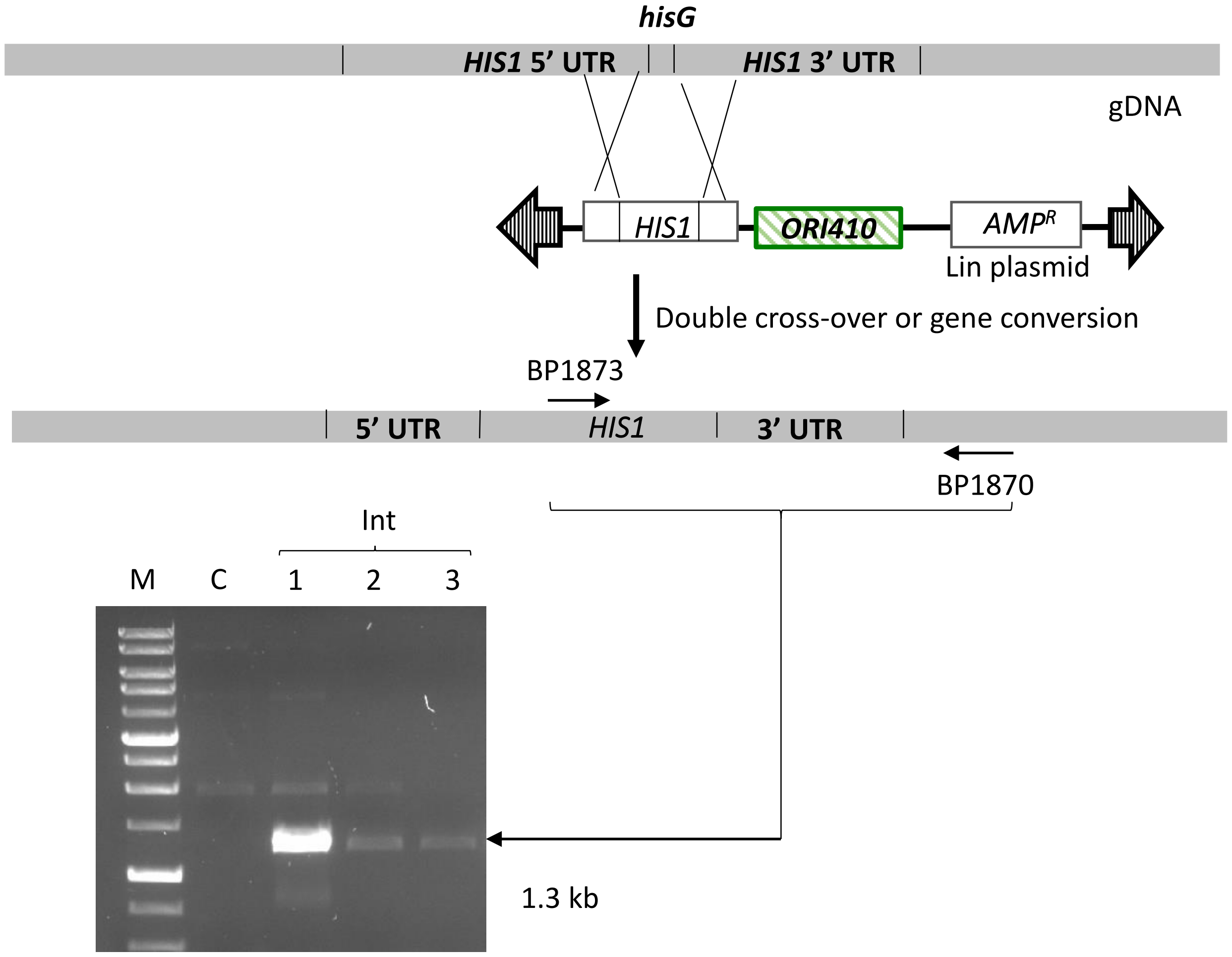

Supplement: FIG S3 [file mSphere.00103-19-sf003.pdf]
