# Supplementary material for: Autonomously Replicating Linear Plasmids That Facilitate the Analysis of Replication Origin Function in Candida albicans
Source: mSphere. 2019 Mar 6;4(2):e00103-19. doi: 10.1128/mSphere.00103-19 (PMC6403455; doi:10.1128/mSphere.00103-19)
Supplement: TABLE S2 [file mSphere.00103-19-st002.pdf]

Table S2.

**pLin-*ORI410*** <br>**Markers**	**Mitotic stability**		
	**1st passage**	**2nd passage**	**3rd passage**
CaHIS1	22 ± 14	25.7 ± 12	27.4 ± 9.9
CdARG4	15.7 ± 3	13.8 ± 2	21.6 ± 10
CmLEU2	29.1 ± 7	16.3 ± 1	20.1 ± 11.6
